# Supplementary material for: Color morphing surfaces with effective chemical shielding
Source: Nat Commun. 2024 May 3;15:3735. doi: 10.1038/s41467-024-48154-y (PMC11068873; doi:10.1038/s41467-024-48154-y)
Supplement: Supplementary file 1 — Supplementary Information [file 41467_2024_48154_MOESM1_ESM.pdf]

Supplementary Information

Color Morphing Surfaces with Effective Chemical Shielding

*Adil Majeed Rather[1], Sravanthi Vallabhuneni[1], Austin J. Pyrch[2], Mohammed Barrubeeah[1], Sreekiran Pillai[1], Arsalan Taassob[1], Felix N. Castellano[2], Arun Kumar Kota[1]**

[1]Department of Mechanical and Aerospace Engineering, North Carolina State University, Raleigh 27695, USA

[2]Department of Chemistry, North Carolina State University, Raleigh 27695-8204, USA

Email: akota2@ncsu.edu

Supplementary Note 1. Spiropyran (SP) to Merocyanine (MC) transformation

SP (closed ring structure) is known to transform into MC (open-ring structure) upon UV irradiation.[1-3] Due to the planar structure and extended π-conjugation of MC, SP to MC photoconversion gives rise to strong absorption, typically in the wavelength range of 550 nm-600 nm.[4,5] Our UV-Vis spectra of polymethyl methacrylate (PMMA) + SP surfaces (Supplementary Fig. 1a) showed no absorbance at 565 nm before UV irradiation (corresponding to SP), but a strong peak was observed at 565 nm after UV irradiation (corresponding to MC). As the UV irradiation time increased, the absorption at 565 nm increased rapidly (Supplementary Fig. 1b). The SP to MC conversion upon UV exposure can be estimated from the absorbance *A* as:

$$\% MC = [(A_{after\ UV} - A_{before\ UV}) / (A_{after\ UV})] \times 100$$

For 70 wt.% PMMA + 30 wt.% SP blends, based on the absorbance measurements (Figure 2b in the main manuscript), we estimated a maximum of 97% conversion of SP to MC after UV exposure (Supplementary Fig. 1c).

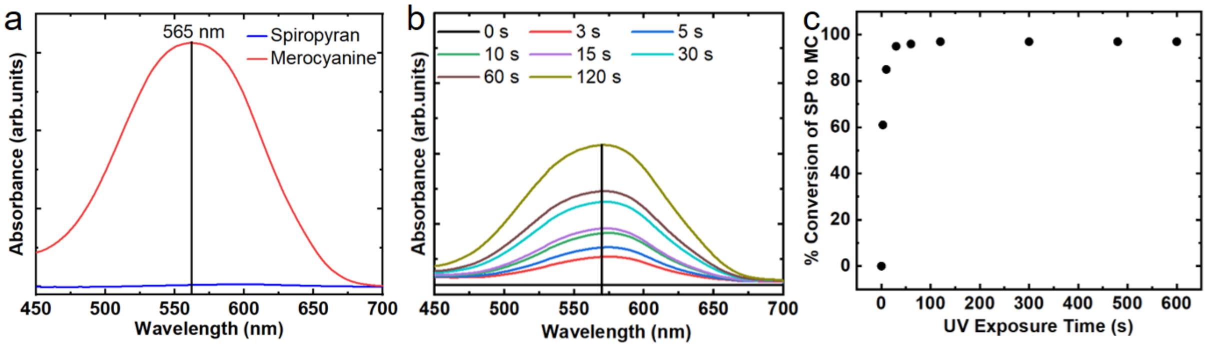

Supplementary Figure 1. UV-Visible absorption spectra of SP to MC transition. a) Absorption spectra of SP (blue) and MC (red). The maximum absorption wavelength (λ_{max}) for MC was at 565 nm. b) Time-dependent change in absorption spectra of SP to MC after continuous irradiation with 365 nm UV light. c) Conversion of SP to MC with increasing UV exposure time.

Supplementary Note 2. Impact of chlorosilanes on color morphing

Among fluorocarbon silanes, chlorosilanes have higher reactivity than ethoxy silanes. However, we chose heptadecafluoro-1, 1, 2, 2-tetrahydrodecyl triethoxysilane (FDTES) instead of heptadecafluoro-1, 1, 2, 2-tetrahydrodecyl trichlorosilane (FDTCS) because hydrolysis of chlorosilanes results in hydrochloric acid, which impedes SP to MC transformation (Supplementary Fig. 2).

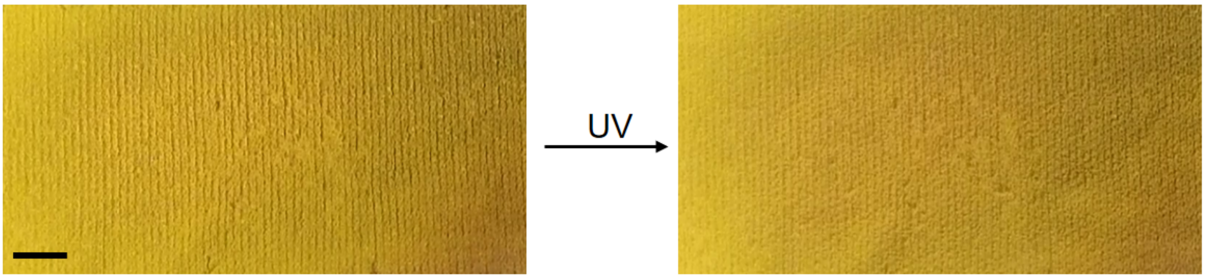

Supplementary Figure 2. Impact of chlorosilanes on color morphing. Images showing no color change after continuous UV irradiation of SP + PMMA + FDTCS surfaces. Scale bar represents 5 mm.

Supplementary Note 3. Color morphing (in solid and liquid states) and liquid repellency in the absence of PMMA and FDTES

To simultaneously achieve color morphing and chemical shielding, an appropriate combination of three materials – a photochromic dye (SP), a low surface energy material (FDTES) and a polymer (PMMA) – is required. The absence of any of these three materials results in poor color morphing and poor liquid repellency.

The as-received SP in the solid-state appears pale yellow; upon exposure to UV, there is no change in color. This indicates that SP by itself does not display photochromic behavior in the solid state (Supplementary Fig. 3a). Upon adding the as-received SP to acetone, the color of the solution turns violet immediately. Upon exposure of SP in acetone solution to UV, there is no further change in color (Supplementary Fig. 3b). The surfaces fabricated by spray coating SP in acetone solutions (without PMMA) show poor color morphing with low color intensity and slow kinetics (Supplementary Fig. 3c and 3d; compare with Fig. 2c in the main manuscript). Furthermore, upon adding the as-received SP and PMMA to acetone, the color of the solution turns violet immediately. Upon exposure of this SP and PMMA in acetone solution to UV, there is no further change in color (Supplementary Fig. 3e).

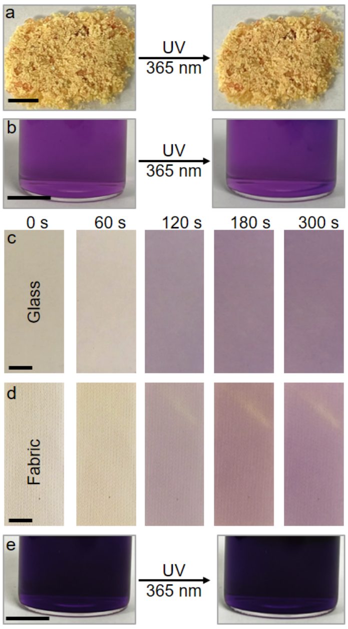

Supplementary Figure 3. Color morphing of SP in solid and liquid state. a) and b) Images showing SP in solid state and as a solution in acetone, respectively, before and after UV exposure. c) and d) Images showing color morphing of SP (without PMMA) spray coated on glass and polyester fabric, respectively. e) Images showing a solution of 70 wt.% PMMA + 30 wt.% SP in acetone before and after UV exposure. Scale bar represents 5 mm.

The surfaces fabricated without FDTES (Supplementary Fig. 4a and 4b) or PMMA (Supplementary Fig. 4c and 4d) display low contact angles, which lead to poor chemical shielding.

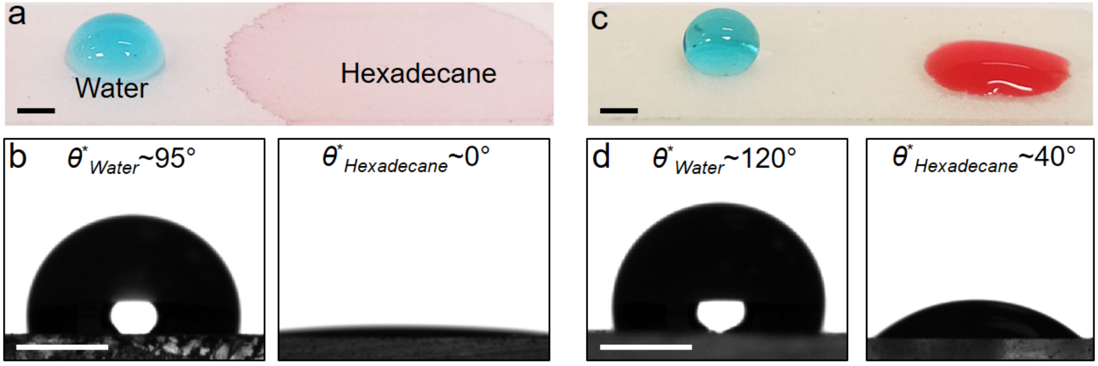

Supplementary Figure 4. Influence of polymer on liquid repellency. a) and b) Color and side-view silhouette images, respectively, of water and hexadecane droplets on PMMA + SP (without FDTES). c) and d) Color and side-view silhouette images, respectively, of water and hexadecane droplets on SP + FDTES (without PMMA). Scale bar is 5 mm for a, c and 1 mm for b, d.

Supplementary Note 4. Reversible color morphing at different temperatures

To demonstrate reversible color morphing at different temperatures, we placed our glass (Supplementary Fig. 5a) and polyester fabric (Supplementary Fig. 5b) coated with PMMA + SP blends at room temperature (~20°C) and at an elevated temperature (60°C). While there was no difference in color morphing (from pale yellow to violet) upon UV exposure at room temperature or 60°C, the reverse color morphing (from violet to pale yellow because of MC to SP transformation via ring-closing) required about 10 h at room temperature and 5 min at 60°C. The half-life $t_{1/2}$ of MC to SP transformation is the time required for 50% MC to transform into SP. For a first order reaction, the half-life is 10% of the time taken for 99% conversion.[6,7] Since MC to SP is a first order reaction,[8-12] we estimated that $t_{1/2} \approx 1$ h at room temperature and $t_{1/2} \approx 0.5$ min at 60°C. Furthermore, the color morphing from pale yellow to violet (in response to UV irradiation) and violet to pale yellow (at room temperature or elevated temperatures) was effective for 10 cycles without loss in photochromism or superomniphobicity (Supplementary Fig. 5c and 5d).

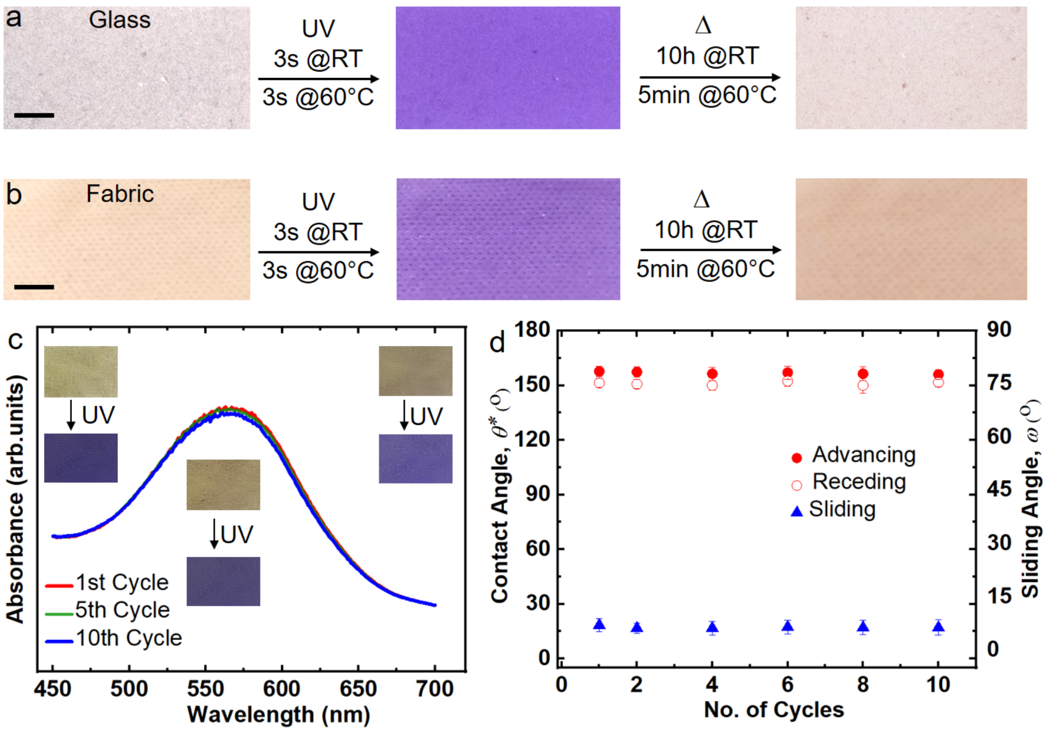

Supplementary Figure 5. Reversible color morphing. a) and b). Images showing the reversible color morphing of 70 wt.% PMMA + 30 wt.% SP surfaces at 60°C on glass and polyester fabric, respectively. Scale bar represents 5 mm. c) UV-Vis absorption spectra indicating effective color morphing for 10 cycles without loss in photochromism. Insets show color morphing upon UV exposure after 1, 5 and 10 cycles. d) Advancing and receding contact angles, as well as sliding angles of hexadecane droplets, indicating effective color morphing for 10 cycles without loss in superomniphobicity. Error bars represent SD.

Supplementary Note 5. Color morphing with different UV lights

Our PMMA + SP surfaces are versatile and can be used with a wide range of UV irradiation. In addition to 365 nm UV irradiation (Fig. 2c, 4c and 4d in the main manuscript), our PMMA + SP surfaces display color morphing (albeit with different kinetics) upon UV irradiation with 254 nm and 385 nm lights (Supplementary Fig. 6a and 6b), as well as sunlight (Supplementary Fig. 6c).

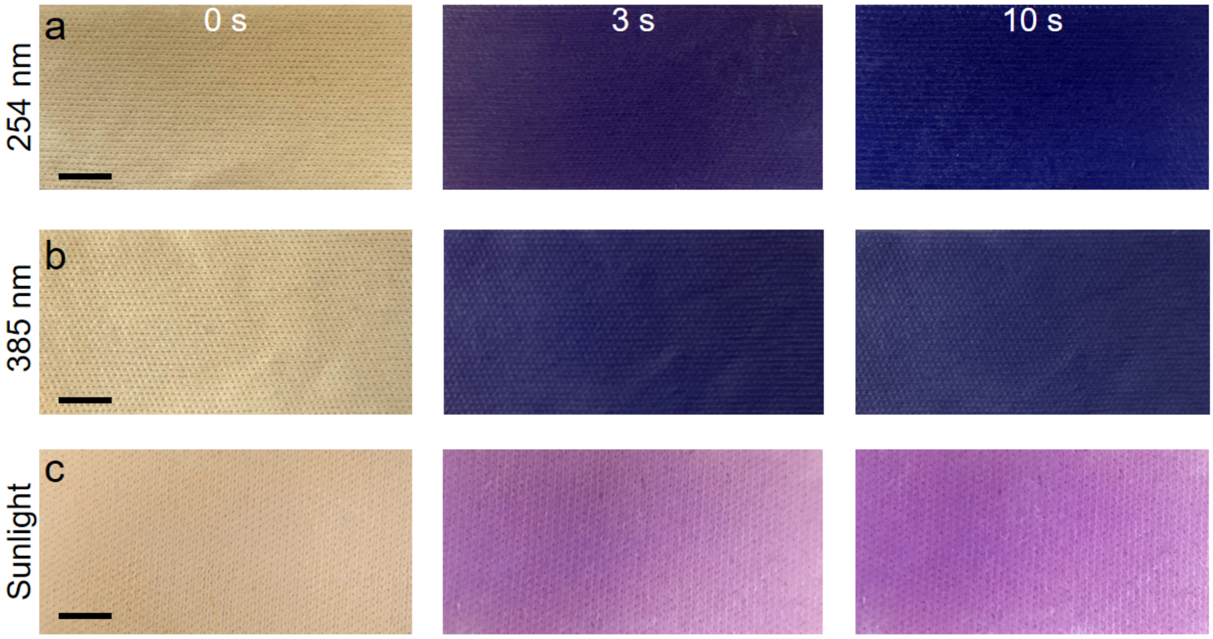

Supplementary Figure 6. Color morphing with different UV lights. a), b) and c). Images showing the color morphing of 70 wt.% PMMA + 30 wt.% SP surfaces upon UV irradiation with 254 nm light, 385 nm light and sunlight, respectively. Scale bar represents 5 mm.

Supplementary Note 6. Surface morphology of color morphing surfaces

We characterized the surface morphology of PMMA + SP blends and PMMA + SP + FDTES blends spray coated on glass and polyester fabric using SEM. There was no noticeable difference in morphology upon addition of FDTES (Supplementary Fig. 7).

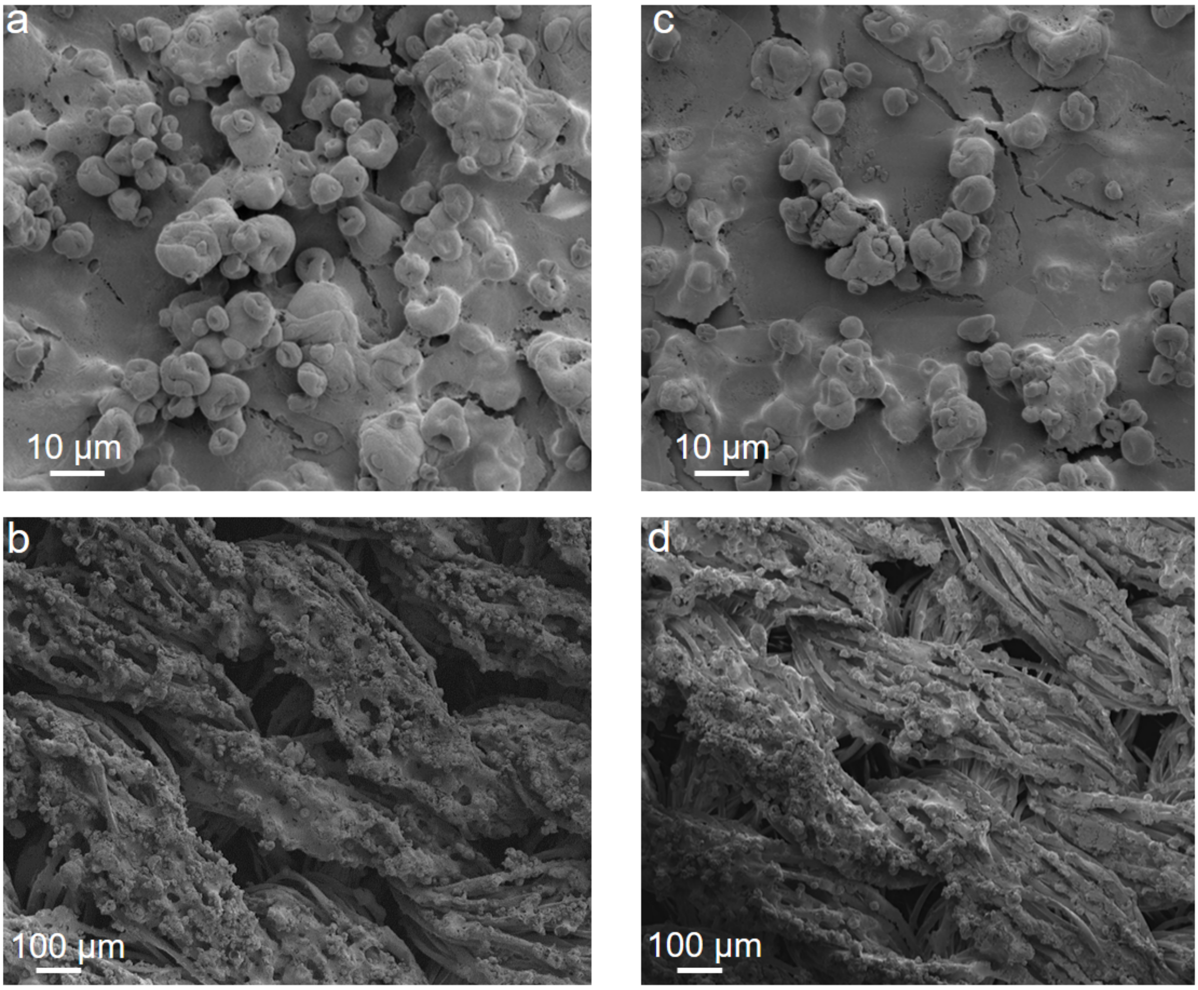

Supplementary Figure 7. Characterization of surface morphology. a) and b) SEM images of glass and polyester fabric, respectively, spray coated with 70 wt.% PMMA + 30 wt.% SP blends. c) and d) SEM images of glass and polyester fabric, respectively, spray coated with PMMA + SP + FDTES blends.

Supplementary Note 7. Estimation of contact angles

When a liquid droplet adopts the Cassie-Baxter state on a textured solid surface, the contact angle θ^* is given by the Cassie-Baxter equation as:

$$\cos\theta^* = f_{sl}\cos\theta - f_{lv} \tag{1}$$

Assuming the surfaces of PMMA + SP + FDTES blends spray coated on glass slides are composed of a hexagonal array of spheres, the Cassie-Baxter equation can be rewritten as:[13]

$$\cos\theta^*_{particle} = -1 + \frac{1}{D^*_{particle}}\left[\frac{\pi}{2\sqrt{3}}(1+\cos\theta)^2\right] \tag{2}$$

On non-textured PMMA + SP + FDTES blend surfaces (prepared via spin coating), we determined the contact angle $\theta = 120°$ for water and $\theta = 80°$ for hexadecane. For $D^*_{particle} = 9$, on surfaces of PMMA + SP + FDTES blends spray coated on glass slides, we estimated the contact angles $\theta^* = 167°$ for water and $\theta^* = 150°$ for hexadecane, which match reasonably well with the experimental values ($\theta^* = 160°$ for water and $\theta^* = 154°$ for hexadecane).

Assuming the surfaces of PMMA + SP + FDTES blends spray coated on polyester fabrics are composed of a hexagonal array of spheres (from spray coating) superimposed on cylindrical fibers (from underlying polyester fabric), the Cassie-Baxter equation can be rewritten as:[13]

$$\cos\theta^*_{hierarchical} = -1 + \frac{1}{D^*_{fiber}}\left[\sin\theta^*_{particle} + \left(\pi - \theta^*_{particle}\right)\cos\theta^*_{particle}\right] \tag{3}$$

For $D^*_{fiber} = 1.3$, on surfaces of PMMA + SP + FDTES blends spray coated on polyester fabrics, we estimated the contact angles $\theta^* = 174°$ for water and $\theta^* = 163°$ for hexadecane which match reasonably well with the experimental values ($\theta^* = 165°$ for water and $\theta^* = 157°$ for hexadecane).

Supplementary Note 8. Estimation of sliding angles

Based on a balance between work done by gravitational force and work expended due to adhesion, the sliding angle ω on a super-repellent surface is given as:[14]

$$\rho g V \approx \gamma_{lv} D_{TCL}(\cos\theta^*_{rec} - \cos\theta^*_{adv}) \quad (4)$$

Here, γ_{lv}, ρ and V are surface tension, density, and volume of the liquid droplet, respectively, and g is the acceleration due to gravity, θ^*_{adv} and θ^*_{rec} are the advancing contact angle and the receding contact angle, respectively, and D_{TCL} is the width of the triple phase contact line perpendicular to the sliding direction. When the shape of the droplet does not deviate significantly from a spherical cap, the width of the triple phase contact line can be computed as:

$$D_{TCL} = 2\cos\left(\bar{\theta}^* - \frac{\pi}{2}\right)\left[\frac{3V}{\pi(2-3\cos\bar{\theta}+\cos^3\bar{\theta})}\right]^{\frac{1}{3}} \quad (5)$$

Here, $\bar{\theta}^*$ is the average contact angle, given as:

$$\bar{\theta}^* = \frac{\cos\theta^*_{adv}+\cos\theta^*_{adv}}{2} \quad (6)$$

The experimentally measured roll off angles of ~20 µL droplets of water and hexadecane are in reasonable agreement with those predicted using equations 4-6 (see Supplementary Table 1).

Supplementary Table 1. Measured and predicted sliding angles of water and hexadecane. NS is no sliding.

	**Water**		**Hexadecane**	
FDTES (wt.%)	ω (measured)	ω (predicted)	ω (measured)	ω (predicted)
0	60°	55°	NS	NS
5	40°	36°	NS	NS
15	6°	4°	NS	NS
25	4°	2°	25°	20°
35	3°	1°	7°	4°
45	3°	1°	7°	3°
60	3°	1°	6°	3°

Supplementary Note 9. FTIR Characterization

Fourier transform infrared spectroscopy (FTIR) was used to characterize the chemical composition of different surfaces. FTIR spectra of PMMA showed absorption peaks around 2951 cm^{-1}, 1721 cm^{-1} and 1159 cm^{-1}, indicating the presence of -C-H, -C=O and -O-CH_3 groups, respectively (Supplementary Fig. 8a).[15,16] FTIR spectra of SP showed absorption peaks around 2964 cm^{-1}, 1609 cm^{-1}, 1511 cm^{-1} and 1267 cm^{-1}, indicating the presence of -C-H, conjugated -C=C-, -NO_2- and -C-N- groups, respectively (Supplementary Fig. 8b).[17,18] FTIR spectra of FDTES showed absorption peaks around 1210 cm^{-1}, 981 cm^{-1}, 736 cm^{-1} and 537 cm^{-1}, indicating the presence of -C-F_x groups (Supplementary Fig. 8c).[19,20] FTIR spectra of 70 wt.% PMMA + 30 wt.% SP showed absorption peaks confirming the coexistence of PMMA and SP (Supplementary Fig. 8d).

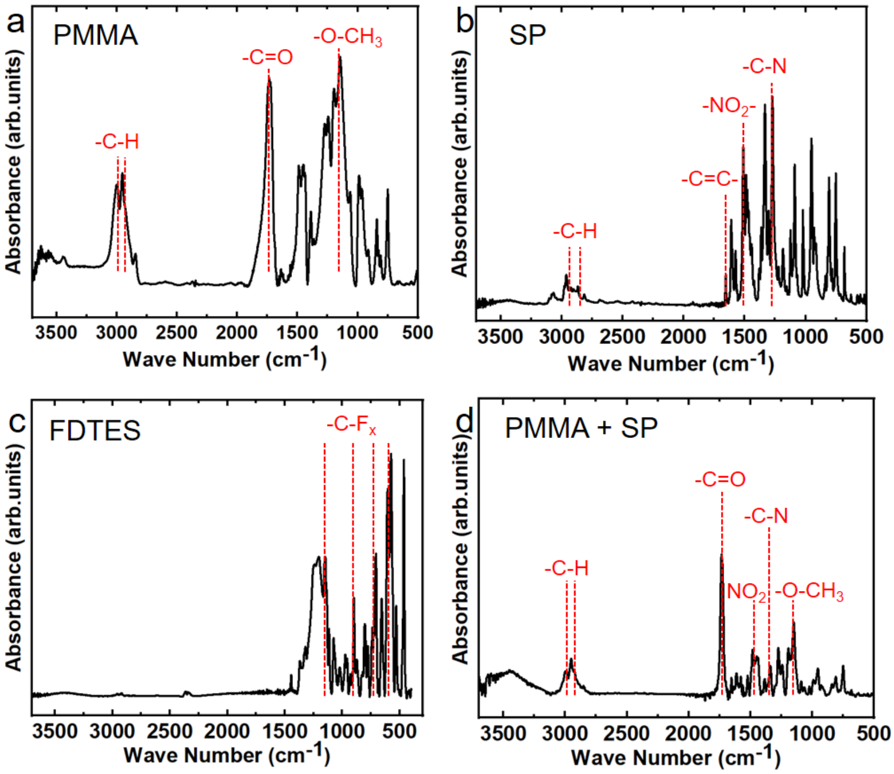

Supplementary Figure 8. FTIR characterization. a), b), c) and d). FTIR spectra of PMMA, SP, FDTES and PMMA + SP, respectively.

Supplementary Note 10. Influence of FDTES on color morphing kinetics

A comparison of the color morphing kinetics of our PMMA + SP + FDTES surfaces (Supplementary Fig. 9) and PMMA + SP surfaces (Fig. 2b in the main manuscript) indicated no significant differences upon addition of FDTES.

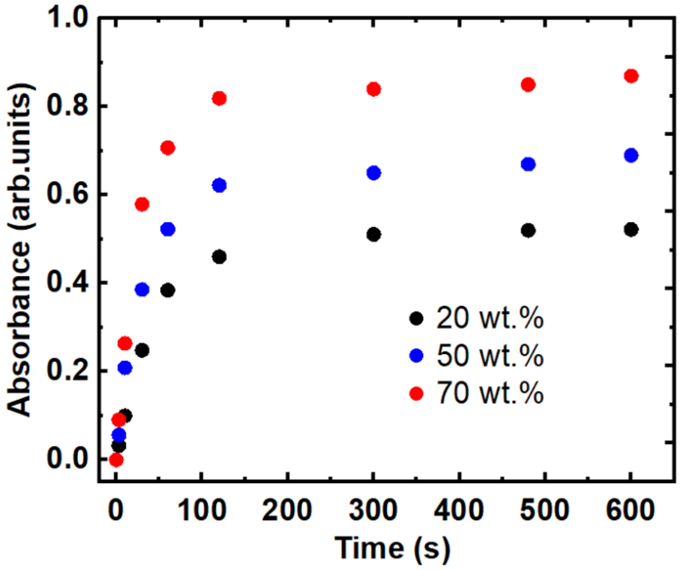

Supplementary Figure 9. Influence of FDTES on color morphing reaction kinetics. Absorbance at 565 nm for our PMMA + SP + FDTES surfaces at different PMMA compositions.

Supplementary Note 11. Breathability of fabrics before and after spray coating

To assess the breathability, we measured the permeability of air through the fabrics, before and after spray coating with PMMA + SP + FDTES blend, using a capillary flow porometer (Quantachrome Porometer 3G). The difference in flow rate of air through the fabrics, before and after spray coating, was insignificant across a spectrum of pressure differentials, indicating that the fabrics retained their breathability even after coating.

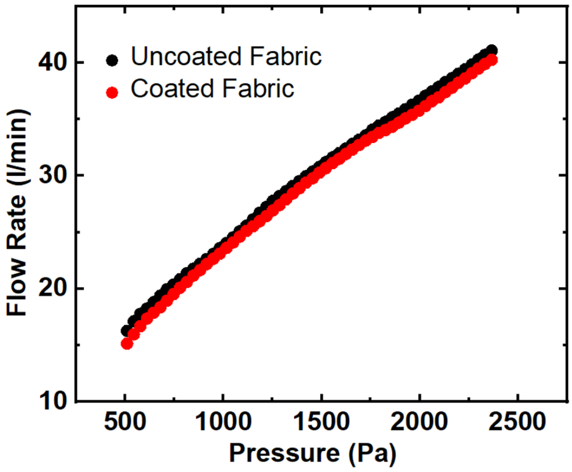

Supplementary Figure 10. Breathability of the fabrics before and after spray coating. Flow rate of air through the fabrics at different pressure differentials.

Supplementary Note 12. Resistance to humid conditions and harsh chemicals

To demonstrate the resistance of our PMMA + SP + FDTES surfaces to humid conditions and harsh chemicals, we exposed them to air with 50% relative humidity, immersion in water and exposure to corrosive liquids with different pH values. The pH of the corrosive liquids was systematically varied from 7 to 1 by adding hydrochloric acid (Fisher) to water and from 7 to 13 by adding sodium hydroxide (Fisher) to water. We measured the advancing and receding contact angles as well as the sliding angles to assess superomniphobicity (Supplementary Fig. 11a, 11b and 11c) and UV-Vis absorption spectra to assess color morphing (Supplementary Fig. 11d, 11e and 11f). Our results indicated no change in superomniphobicity or color morphing even after continuous exposure to air with 50% relative humidity for 10 days, continuous immersion in water for 10 days, and exposure to corrosive liquids with different pH for 1 h.

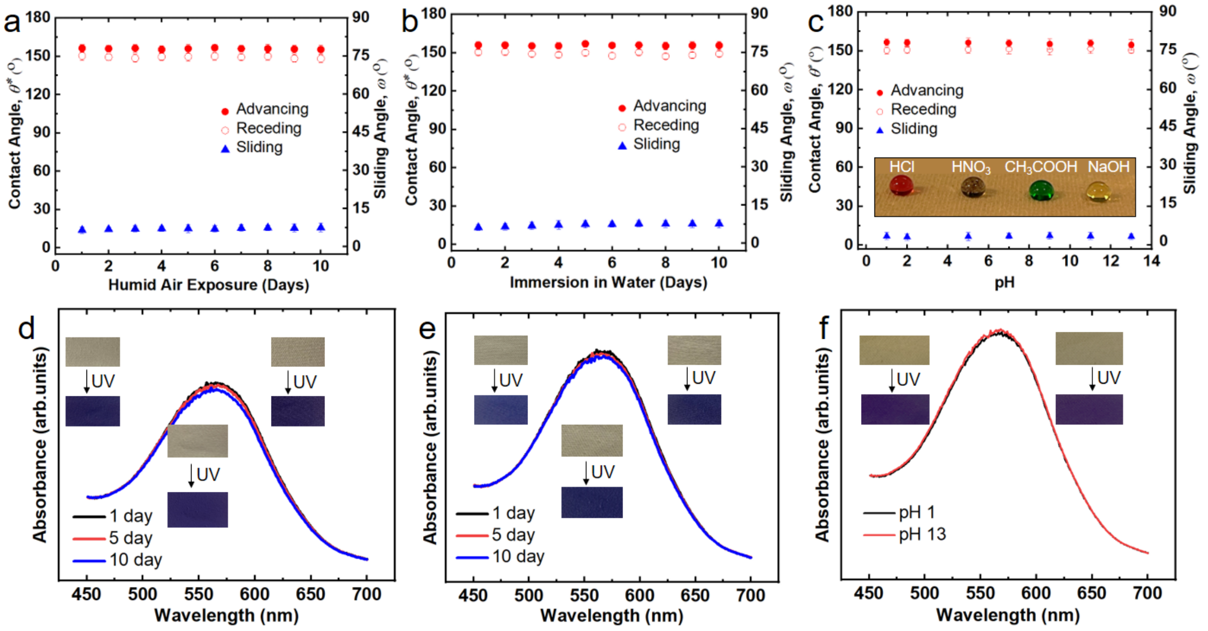

Supplementary Figure 11. Resistance of color morphing superomniphobic surfaces to humid conditions and harsh chemicals. a), b) and c) Advancing and receding contact angles, as well as sliding angles of hexadecane on color morphing superomniphobic surfaces after humid air exposure, immersion in water and exposure to liquids with different pH values, respectively. Inset in c shows droplets of hydrochloric acid (HCl (5M); red), nitric acid (HNO_3 (5M); grayish black), acetic acid (CH_3COOH; green) and sodium hydroxide (NaOH (5M); yellow) adopting the Cassie-Baxter state and beading up on PMMA + SP + FDTES surfaces. Error bars represent SD. d), e) and f) UV-Vis absorption spectra of color morphing superomniphobic surfaces after humid air exposure, immersion in water and exposure to liquids with different pH values, respectively.

Supplementary Note 13. Contact angles and sliding angles on different substrates

The advancing and receding contact angles and sliding angles of ~20 μL droplets of water and hexadecane on different substrates are summarized in Supplementary Table 2. Both water and hexadecane adopted the Cassie-Baxter state with contact angles θ^* >150° and sliding angles ω < 10° on all the substrates.

Supplementary Table 2. Advancing and receding contact angles, and sliding angles of color morphing superomniphobic surfaces on different substrates. HD is hexadecane.

**Substrate**	θ^*_{adv} (°)		θ^*_{rec} (°)		ω (°)	
	**Water**	**HD**	**Water**	**HD**	**Water**	**HD**
Paper	158±2	155±2	153±1	148±2	5±2	8±1
Acrylic	157±2	154±2	154±1	146±1	4±1	8±1
PET	158±1	152±1	154±2	143±2	5±1	7±2
Aluminum	159±1	153±2	155±2	146±1	6±2	7±1
Copper	156±2	152±2	150±2	145±2	6±1	9±1
Tin	157±2	153±1	151±1	144±2	5±2	8±1

Supplementary Note 14. Contact angles and sliding angles of superomniphobic surfaces with different photochromic and thermochromic pigments

The advancing and receding contact angles and sliding angles of ~20 μL droplets of water and hexadecane in the Cassie-Baxter state on superomniphobic surfaces with different photochromic and thermochromic pigments (Supplementary Table 3).

Supplementary Table 3. Advancing and receding contact angles and sliding angles of water and hexadecane on superomniphobic surfaces with different photochromic and thermochromic pigments. HD is hexadecane, Y is yellow, G is green, P is purple, B is blue, W is white, LG is light green, and LB is light brown.

Surfaces	θ^*_{adv} (°)		θ^*_{rec} (°)		ω (°)	
	**Water**	**HD**	**Water**	**HD**	**Water**	**HD**
Y to G	157±2	154±2	152±1	147±2	4±1	7±1
Y to R	158±1	155±2	153±1	147±1	5±1	8±1
Y to P	156±2	153±1	152±2	145±1	4±2	8±1
B to V	158±1	152±1	154±2	143±2	5±1	8±2
R to W	157±2	153±2	151±2	146±2	5±1	7±2
LB to LG	158±1	154±1	152±1	146±1	4±2	7±1

Furthermore, the color morphing was effective for hundreds of cycles without loss in photochromism or superomniphobicity (Supplementary Fig. 12).

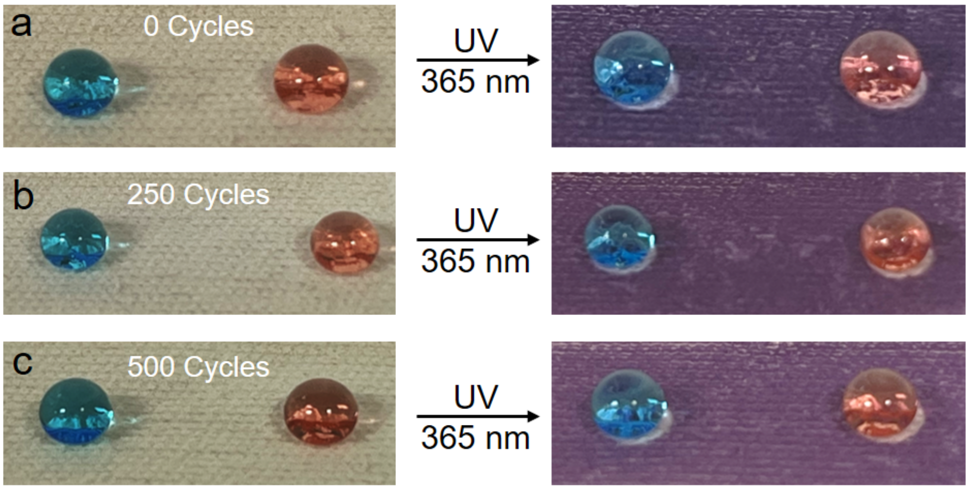

Supplementary Figure 12. Reversible color morphing. a) b) and c) Droplets of water (blue) and hexadecane (red) adopting the Cassie-Baxter state and beading up on color morphing superomniphobic surfaces after 0, 250 and 500 color morphing cycles, respectively.

Supplementary Note 15. Durability of color morphing superomniphobic surfaces

We investigated the retention of superomniphobicity on our color morphing superomniphobic surfaces after water and hexadecane droplets slid past the surface. Our results indicated no change in advancing and receding contact angles and sliding angles of hexadecane droplets (~20 μL) on our color morphing superomniphobic surfaces even after 10,000 droplets slid past the surface (Supplementary Fig. 13a and 13b). While our surfaces can retain superomniphobicity against liquids, they do not have sufficient durability to withstand abrasion against solids.

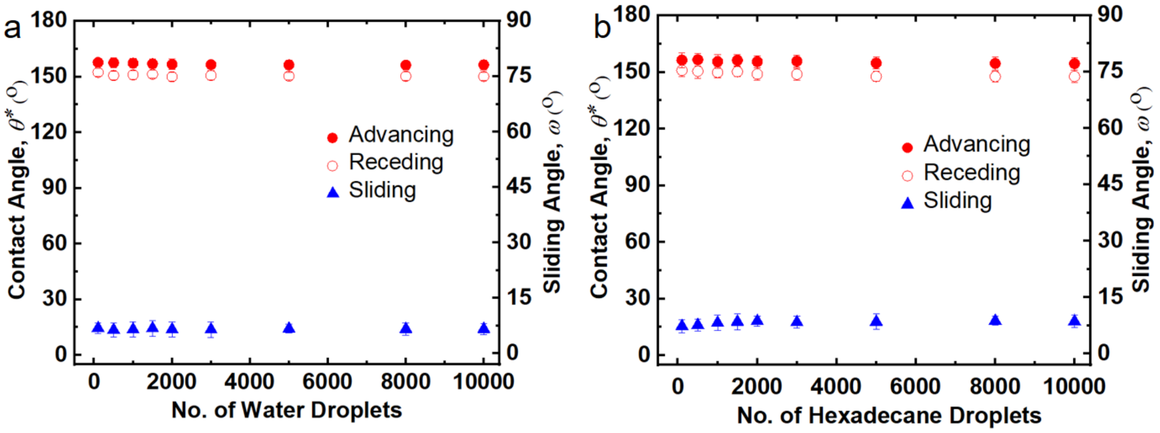

Supplementary Figure 13. Durability of color morphing superomniphobic surfaces. a) and b) Advancing and receding contact angles as well as sliding angles of hexadecane droplets on our color morphing superomniphobic surface as a function of the number of water and hexadecane droplets, respectively, sliding past the surface. Error bars represent SD.

Supplementary References

1 Kaiser, C., Halbritter, T., Heckel, A. & Wachtveitl, J. Thermal, photochromic and dynamic properties of water-soluble spiropyrans. *ChemistrySelect* **2**, 4111-4123 (2017).
2 Zhi, J. F., Baba, R., Hashimoto, K. & Fujishima, A. Photoelectrochromic properties of a spirobenzopyran derivative. *Journal of Photochemistry and Photobiology A: Chemistry* **92**, 91-97 (1995).
3 Liu, C., Yang, D., Jin, Q., Zhang, L. & Liu, M. A chiroptical logic circuit based on self-assembled soft materials containing amphiphilic spiropyran. *Advanced Materials* **28**, 1644-1649 (2016).
4 Buback, J. *et al.* Ultrafast bidirectional photoswitching of a spiropyran. *Journal of the American Chemical Society* **132**, 16510-16519 (2010).
5 Chernyshev, A. V. *et al.* Metal complexes of new photochromic chelator: Structure, stability and photodissociation. *Journal of Photochemistry and Photobiology A: Chemistry* **265**, 1-9 (2013).
6 Levenspiel, O. *Chemical reaction engineering.* (John wiley & sons, 1998).
7 Fogler, H. S. *Essentials of chemical reaction engineering: essenti chemica reactio engi.* (Pearson Education, 2010).
8 Richert, R. & Heuer, A. Rate-memory and dynamic heterogeneity of first-order reactions in a polymer matrix. *Macromolecules* **30**, 4038-4041 (1997).
9 Ren, J. & Tian, H. Thermally stable merocyanine form of photochromic spiropyran with aluminum ion as a reversible photo-driven sensor in aqueous solution. *Sensors* **7**, 3166-3178 (2007).
10 Bao, L.-h., Sun, J.-x. & Li, Q. Synthesis and properties of waterborne polyurethane containing spiropyran groups. *Journal of Polymer Research* **21**, 1-7 (2014).
11 Wang, J. *et al.* Altering the Properties of Spiropyran Switches Using Coordination Cages with Different Symmetries. *Journal of the American Chemical Society* **144**, 21244-21254 (2022).
12 Zhang, Y. *et al.* Synthesis and photoswitchable amphiphilicity and self-assembly properties of photochromic spiropyran derivatives. *Journal of Materials Chemistry C* **8**, 13676-13685 (2020).
13 Kota, A. K., Li, Y., Mabry, J. M. & Tuteja, A. Hierarchically structured superoleophobic surfaces with ultralow contact angle hysteresis. *Advanced materials* **24**, 5838-5843 (2012).
14 Furmidge, C. Studies at phase interfaces. I. The sliding of liquid drops on solid surfaces and a theory for spray retention. *Journal of colloid science* **17**, 309-324 (1962).
15 Sain, S. *et al.* Synthesis and characterization of PMMA-cellulose nanocomposites by in situ polymerization technique. *Journal of Applied Polymer Science* **126**, E127-E134 (2012).
16 Ramesh, S., Leen, K. H., Kumutha, K. & Arof, A. FTIR studies of PVC/PMMA blend based polymer electrolytes. *Spectrochimica Acta Part A: Molecular and Biomolecular Spectroscopy* **66**, 1237-1242 (2007).
17 Bao, B., Fan, J., Wang, W. & Yu, D. Photochromic cotton fabric prepared by spiropyran-ternimated water polyurethane coating. *Fibers and Polymers* **21**, 733-742 (2020).
18 Ahmadi, S., Nasiri, M. & Pourrajab Miandoab, A. Synthesis and characterization of a pH and photoresponsive copolymer of acrylamide and spiropyran. *Polymers for Advanced Technologies* **31**, 2545-2551 (2020).
19 Molina, R., Teixidó, J. M., Kan, C.-W. & Jovančić, P. Hydrophobic coatings on cotton obtained by in situ plasma polymerization of a fluorinated monomer in ethanol solutions. *ACS applied materials & interfaces* **9**, 5513-5521 (2017).

20 Mihály, J., Sterkel, S., Ortner, H. M., Kocsis, L., Hajba, L., Furdyga, É., & Mink, J. FTIR and FT-Raman spectroscopic study on polymer based high pressure digestion vessels. *Croatica chemica acta* **79**, 497-501 (2006).
