# Supplementary material for: Color morphing surfaces with effective chemical shielding
Source: Nat Commun. 2024 May 3;15:3735. doi: 10.1038/s41467-024-48154-y (PMC11068873; doi:10.1038/s41467-024-48154-y)
Supplement: Supplementary file 3 — Description of Additional Supplementary Files [file 41467_2024_48154_MOESM3_ESM.pdf]

Description of Additional Supplementary Files

Supplementary Movie Legends

Supplementary Movie 1. This video illustrates the sliding of ~20 μL water (blue) and hexadecane (red) droplets on a fabric spray coated with PMMA + SP + FDTES blend, before and after exposure to 365 nm UV light. The surface was tilted by 7˚ relative to the horizontal.

Supplementary Movie 2. This video illustrates the camouflage of a fabric spray coated with PMMA + SP + FDTES blend upon UV exposure. The coated fabric was placed on a pale-yellow background and gradually moved to a violet background, while simultaneously exposing the fabric to UV light. As the coated fabric transitioned to the violet background, it rapidly turned into the violet color and blended with the background.

Supplementary Movie 3. This high-speed video (500 frames per second) illustrates the bouncing of ~10 μL water (blue) and hexadecane (red) droplets on a fabric spray coated with PMMA + SP + FDTES blend.

Supplementary Movie 4. This video illustrates the immersion of a fabric spray coated with PMMA + SP blend (left) and PMMA + SP + FDTES blend (right) in 6M Sulfuric acid solution, followed by UV exposure, indicating that PMMA + SP + FDTES surface retains color morphing, but PMMA + SP surface does not.

Supplementary Movie 5. This video illustrates the immersion of a fabric spray coated with PMMA + SP + FDTES blend in 30% H_2O_2 solution, followed by UV exposure, indicating no difference in color morphing for areas exposed and unexposed to 30% H_2O_2 solution.

Supplementary Movie 6. This video illustrates the sliding of ~20 μL water (blue) and hexadecane (red) droplets on superomniphobic surfaces with different photochromic pigments, before and after exposure to 365 nm UV light. The surface was tilted by 7˚ relative to the horizontal.

Supplementary Movie 7. This video illustrates the sliding of ~20 μL water (blue) and hexadecane (red) droplets on superomniphobic surfaces with different thermochromic pigment, before and after heating. The surface was tilted by 7˚ relative to the horizontal.

Supplementary Movie 8. This video illustrates a photochromic superomniphobic fabric with "NC STATE" pattern, before and after exposure to 365 nm UV light.
